# Emergency Medical Services Data for Cardiovascular Disease Surveillance, Program Planning, and Evaluation in Maine

**Published:** 2008-03-15

**Authors:** Katie A Meyer, Kathy Decker, Cynthia A Mervis, Danielle Louder, Jay Bradshaw, Shannon DeVader, Debra Wigand

**Affiliations:** Division of Chronic Disease/Maine Center for Disease Control and Prevention. The author also is affiliated with the University of Southern Maine, Department of Applied Medical Sciences, Portland, Maine; Maine Center for Disease Control and Prevention, Cardiovascular Health Program, Augusta, Maine, and University of Southern Maine, Department of Applied Medical Sciences, Portland, Maine; Maine Center for Disease Control and Prevention, Cardiovascular Health Program, Augusta, Maine, and University of Southern Maine, Department of Applied Medical Sciences, Portland, Maine; Maine Center for Disease Control and Prevention, Cardiovascular Health Program, Augusta, Maine, and Medical Care Development, Augusta, Maine; Maine Emergency Medical Services, Department of Public Safety, Augusta, Maine; Centers for Disease Control and Prevention, Council of State and Territorial Epidemiologists, Applied Epidemiology Fellowship, Augusta, Maine; Maine Center for Disease Control and Prevention, Cardiovascular Health Program, Augusta, Maine

## Abstract

Rapid access to medical treatment is a key determinant of outcomes for cardiovascular events. Emergency medical services (EMS) play an important role in delivering early treatment for acute cardiovascular events. Attention has increased on the potential for EMS data to contribute to our understanding of prehospital treatment. Maine recently began to explore the possible role of EMS data in cardiovascular disease surveillance and cardiovascular health program planning and evaluation. We describe the Maine EMS data system, discuss findings on ease of data use and data quality, provide a sample of findings, and share how we plan to use EMS data for program planning and evaluation of community-level interventions and to partner with EMS provider organizations to improve treatment. Our objective is to increase understanding of the promise and limitations of using EMS data for cardiovascular disease surveillance and program planning and evaluation.

## Background

Rapid access to medical care after a major cardiovascular event decreases morbidity and mortality. The chain-of-survival framework, originally described by Cummins, delineates the components upon which timely treatment depends, including awareness of signs and symptoms; care seeking; and aspects of emergency medical dispatch, emergency medical services (EMS), and emergency department (ED) and hospital systems (1). The best outcomes occur with a timely and well-coordinated response and the use of a systems approach to care (2).

The importance of early medical response to positive cardiovascular event outcomes is reflected in the Centers for Disease Control and Prevention's (CDC's) Division for Heart Disease and Stroke Prevention's priority areas. These include recognizing the signs of heart attack (myocardial infarction) and stroke, increasing the number of calls to 9-1-1, and improving emergency response. The relationship between early treatment and positive outcomes has spurred state and national interest in supplementing data from EDs and hospitals with EMS data for public health surveillance, program planning and evaluation, and quality of care assessment. Despite their importance, EMS data are not universally available to state public health professionals, and data collection is not standardized across states. To improve and standardize EMS data, 49 states, including Maine, have agreed to participate in the National EMS Information System (NEMSIS), which seeks to ensure consistent and valid reporting of a standard set of EMS data indicators and to create a national EMS data set, representing the time from a 9-1-1 telephone call through arrival at a hospital (3).

The Maine Cardiovascular Health Program (MCVHP) of the Maine Center for Disease Control and Prevention has begun to explore the use of EMS data for cardiovascular disease (CVD) surveillance, program planning, and evaluation. In this report, we describe the Maine EMS data system, present basic descriptive findings, discuss ways the data can contribute to the efforts of the MCVHP and its partners, and outline challenges and limitations. Our objective is to contribute to the growing understanding of how state EMS data can be used for CVD surveillance and cardiovascular health program planning and evaluation.

## The Maine EMS Data System

In Maine, legislatively mandated EMS data are collected from all EMS providers on standard run-report forms and submitted monthly to a data-processing organization, where they are combined into a database. To ensure compliance with the Health Insurance Portability and Accountability Act of 1996 (HIPAA), submitted forms omit patient names and addresses. Maine EMS, a bureau within the Department of Public Safety, maintains the database. The system is funded through a combination of state funds, grants, and license and examination fees. Maine EMS shares the data with the MCVHP free of charge.

EMS personnel complete a run report for each service call they receive. The run report includes fields for service (e.g., service number, run date), patient (e.g., date of birth, sex, town of residence), type of run or injury (e.g., primary problem), times and odometer readings (e.g., time call received, time arrived on scene, time left scene, time arrived at destination), assessment of patient at scene and en route (e.g., pulse, blood pressure), and treatments and mutual aid (e.g., defibrillation, mutual aid service number). The run report includes a list of possible medical reasons for the call. EMS personnel indicate by checkbox what they believe most accurately describes the type of problem experienced by the patient; possible CVD events are listed as "cardiac" or "CVA" (cerebrovascular accident).

## Analysis of Maine EMS Data

Here we present our initial analyses, limited to the 75,085 EMS emergency transport runs with hospital destinations during 2000 through 2004 for which the cardiac checkbox was marked. (Data on CVA events will be reported elsewhere.) There was not a substantial amount of missing or illogical data for variables used in our analyses: 2.2% of run reports were missing data on birth date; 0.8%, sex; 0.4%, age; and 0.3%, residence.

The most significant challenge in working with the EMS data was creating an event-level data set from the original run-report data. Multiple run reports may represent a single event — each EMS crew involved in an event will complete a separate run report. Termed *ambulance assist*, care provided by multiple EMS crews could include crews with different levels of training and certification (e.g., basic life support, advanced life support) or a transfer of crew members or patients between ambulances en route to the final destination. The EMS data system lacks unique personal identifiers; run reports for the same event, therefore, must be identified using other variables.

We first restricted the run-report data to runs for which the cardiac checkbox was marked. We then used deterministic linkage methodology based on run date; patient date of birth or age, sex, and town of residence; hospital destination; ambulance assist information; and documented times to identify unique cardiac events (rather than cardiac runs). Our final event-level data set included 71,432 cardiac events. Of these, 67,794 (94.9%) involved a single run report, 3623 (5.1%) involved two run reports, and 15 involved three run reports.

We calculated event-level response and total call-time intervals (in minutes). We defined *response time* as the interval starting with the notification of the ambulance unit by dispatch (*call time*) and ending with the unit's arrival on scene (*scene arrival time*). *Total call time* was defined as the interval starting with call time and ending with the unit's arrival at the hospital destination (*destination time*). For ambulance assists, we used the earliest time listed for each of the call, scene arrival, and destination times. Sensitivity analysis revealed similar statistics when we defined the interval as the earliest call time and the latest destination time listed. We excluded implausibly low and high intervals: negative or zero-valued response times (1.8% of events), greater than 90-minute response times (<0.01% of events), and greater than 6-hour total call times (0.02%).

**Figure 1:**
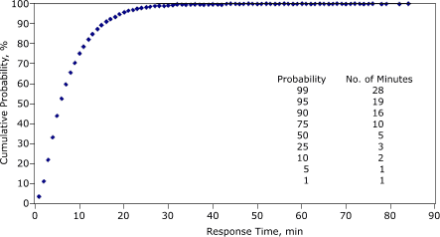
Cumulative probability distribution for response time (minutes) for cardiac-related events, Maine EMS, 2000–2004. Response time is defined as the interval starting with the notification of the EMS unit by dispatch and ending with the unit's arrival on scene. Response time ranged from 1 minute to 84 minutes. EMS indicates emergency medical services.

**Figure 2:**
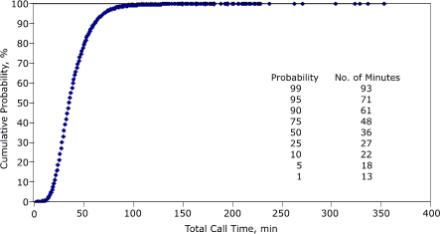
Cumulative probability distribution for total call time (minutes) for cardiac-related events, Maine EMS, 2000–2004. Total call time was defined as starting with notification of the ambulance unit by dispatch and ending with the unit's arrival at the destination. Total call time ranged from 3 minutes to 353 minutes. EMS indicates emergency medical services.

Figure 1 displays the cumulative probability distribution for response time. Twenty-five percent of response times were within 3 minutes, 50% within 5 minutes, and 75% within 10 minutes. Sixty-six percent of response times were within 8 minutes, a referenced standard for EMS (4). Figure 2 displays the cumulative probability distribution for total call time. Twenty-five percent of cardiac events had a total call time within 27 minutes, 50% within 36 minutes, and 75% within 48 minutes. Eighty-nine percent of total call times were within 60 minutes, a referenced standard for the amount of time from symptom initiation to treatment for acute myocardial infarction (5). A 60-minute total call time corresponding to a 60-minute symptom-to-treatment window would require the patient to respond immediately to symptoms (by calling 9-1-1), the EMS dispatcher to transfer the call immediately to an EMS unit, and the hospital to provide care to the patient immediately upon arrival. We assume that 89% of total call times recorded in our data set did not meet these criteria; the 11% of total time calls that exceeded 60 minutes would not even be eligible for achieving that goal.

We then examined how the EMS cardiac checkbox might correspond to more rigorously defined diagnoses. The Table compares rates of EMS cardiac events to rates of hospital discharge, using different sets of cardiac-related primary discharge diagnoses, as defined by the* International Classification of Diseases, Ninth Revision, Clinical Modification* (6). Maine's hospital discharge data set includes all inpatient discharges from Maine's nonfederal hospitals. From birth to 44 years, the rate of EMS cardiac events was 22.3 per 10,000 population. This rate exceeds the rate for even the broadest CVD-related diagnostic category, cardiovascular disease (17.2 per 10,000 population). For people aged 75 years or older, we observed the reverse — the rate of EMS cardiac events (38.0 per 10,000 population) was far below the hospital discharge rate for the most narrowly defined diagnosis, acute myocardial infarction (217.0 per 10,000 population).

These findings are compelling but uncertain. The high EMS rate among people younger than 45 could signify that EMS personnel are more likely to erroneously identify a cardiac event among younger people or that the prehospital death rate is higher for this age group. The low rate of cardiac events among the elderly could signify that more people are transporting themselves to the hospital or that EMS personnel are less likely to use the cardiac checkbox for cardiovascular-related events in this population. In any event, these results underscore the lack of clarity for the clinical entity to which the EMS checkboxes correspond.

## EMS Data for Program Planning and Evaluation

EMS data can contribute to program activities of the MCVHP and other state partners in several ways. In addition to providing surveillance of EMS use and response and total call times, a planned linkage with hospital discharge and outpatient ED data would further illustrate the magnitude and characteristics of the population that self-transports to the ED. The linkage would allow the MCVHP and its partners to better focus on educating the public about the importance of using EMS during a cardiac event (7). Analysis of EMS data on prehospital recognition and treatment, such as data on the use of medication, cardiac monitoring, and defibrillation, could enable the MCVHP, in partnership with Maine EMS, to plan activities for improving the quality of EMS in Maine.

The EMS system provides a data source to evaluate activities related to emergency response. The Maine HeartSafe Community Initiative (www.healthymainepartnerships.org/mcvhp/heartsafe.aspx) is a collaboration between Maine EMS and the MCVHP. The initiative was designed with the immediate goal of recognizing EMS providers for their contribution to local emergency response and providing opportunities for enhancing or increasing EMS capabilities. A secondary goal includes enhancing education on awareness of the signs and symptoms of heart attack and stroke through community partnerships (8).

Available to all services in Maine, the HeartSafe Community Initiative includes 24 EMS providers (of 284 providers in Maine), covering more than 139 communities (including towns, cities, and one university), and representing more than 405,000 residents and five of Maine's six EMS regions. The regions encompass different service area characteristics, such as catchment area size and population density. Services apply to participate in the initiative, completing a self-assessment and inventory of their capacity. The services within each region include a range of HeartSafe designation levels. Services apply for a basic, silver, gold, or platinum designation on the basis of their current capacity to meet program criteria; services that are permitted or licensed at the paramedic level receive the gold or platinum designations. The initiative recognizes services that have met initiative requirements and developed an evaluation and improvement plan. Recognized services reapply to the initiative every 2 years. The application process provides a mechanism to ensure maintenance of program criteria and an opportunity to measure enhanced EMS capacity. The MCVHP maintains a participant database, which is used for technical assistance and evaluation. Ultimately, EMS data can be used to explore whether participation in the initiative improves EMS capacity and quality or increases use by the community.

Another EMS-related project in the state is led by the Maine Quality Forum (MQF), created in 2003 by Governor John Baldacci and the state legislature to ensure the delivery of high-quality health care throughout the state. The manager of the MCVHP (D.W.) is a member of the MQF's executive committee, and MCVHP staff led the MQF's community engagement component. The MQF's In a Heartbeat program was created to track medical care for ST-segment elevation acute myocardial infarctions (STEMIs) through the EMS, ED, and hospital systems. The program involves developing and implementing statewide community engagement initiatives and regionally appropriate response and treatment plans as well as collecting and analyzing evidence-based metrics for STEMI. The MQF chose to focus on STEMIs because STEMIs respond particularly well to early treatment with therapeutic approaches that are available in all of Maine's EDs (9). In a Heartbeat will directly link EMS data to ED and hospital data, tracking individuals through the health care system. As part of the project, the MQF is developing an EMS quality-improvement tool and providing 12-lead electrocardiography training to EMS staff throughout the state.

The MCVHP has also facilitated Stroke Care in Maine (www.mcd.org/registrations_INACTIVE.asp), a statewide effort to initiate a collaborative system of care for stroke patients, and Maine is one of eight states involved in a regional system of stroke care, the NorthEast Cerebrovascular Consortium (www.thenecc.org). Both initiatives are based on the blueprint recommendations released in 2005 by the American Heart Association, the American Stroke Association, and the Brain Attack Coalition (2,10), and both include the following stroke system components: community engagement; prehospital, acute, and subacute care; rehabilitation; and quality improvement. EMS data will be vital in measuring the efficacy of the initiatives and planning the next steps.

## Challenges and Limitations

EMS data have important limitations. The lack of unique personal identifiers challenged our creation of event-level data and will hinder our ability to link with certainty EMS data to hospital discharge and outpatient ED data. Linking EMS data to hospital discharge and outpatient ED data would further our understanding of another limitation — the lack of clarity on the cardiac checkbox — both in terms of the clinical diagnoses to which the cardiac checkbox corresponds and the degree to which EMS personnel can accurately identify cardiovascular events. Another limitation is the lack of dispatch data in the EMS data system in Maine, which prevented our including the time from calling 9-1-1 in our analysis. In addition, the lack of standards for EMS data collection, analysis, and presentation makes comparisons to other EMS data reports difficult.

## Conclusion

EMS data offer a unique perspective on acute cardiovascular events, a perspective valuable to understanding emergency response within states and to planning improvements in the timeliness and quality of emergency response. Analysis of EMS data has expanded our thinking about how emergency response can be studied and evaluated in Maine. The data already have contributed to our knowledge of emergency response, particularly our knowledge of response and total call times.

Further study is needed to more fully evaluate the quality of EMS data and identify ways to capture their full utility. Linking EMS data to hospital discharge and ED outpatient data would further our understanding of self-transport, the degree to which EMS personnel can accurately identify cardiovascular events in the field, and to which diagnoses the cardiac checkbox corresponds.

## Acknowledgments

This report was supported in part by CDC Cardiovascular Health Program Cooperative Agreement U50/CCU121347 and by an appointment to the Applied Epidemiology Fellowship Program administered by the Council of State and Territorial Epidemiologists (CSTE) and funded by CDC Cooperative Agreement U60/CCU007277.

## Figures and Tables

**Table.** Rates per 10,000 Population of Cardiac-Related Events Using EMS and Hospitalization Data, Maine, 2000–2004

Variable	EMS Cardiac Events^a^	Hospitalization^b^			

		Acute MI	Coronary Heart Disease	Diseases of the Heart	Cardiovascular Disease
**Age group, y**					
0-44	22.3	3.7	7.4	13.8	17.2
45-64	108.0	46.8	118.3	173.0	211.2
65-74	276.5	121.6	317.4	560.2	709.9
≥75	38.0	216.8	413.0	944.7	1225.4
**Sex, age-adjusted (crude)**					
Male	101.2 (102.4)	44.7 (46.4)	103.9 (109.2)	176.3 (181.3)	220.2 (225.6)
Female	88.8 (109.6)	24.5 (31.3)	53.9 (67.7)	111.9 (142.2)	144.3 (183.5)
**Overall age-adjusted (crude)**	94.7 (106.1)	33.9 (38.6)	77.0 (87.9)	141.4 (161.3)	178.9 (204.0)

EMS indicates emergency medical services; MI, myocardial infarction.

a EMS personnel complete a run report for each service call they receive. The run report includes a list of possible medical reasons for the call. EMS personnel indicate what they believe most accurately describes the type of problem experienced by the patient; possible CVD events are listed as "cardiac" and "CVA" (cerebrovascular accident).

b Hospitalizations include admissions of Maine residents with a primary discharge diagnosis of acute myocardial infarction (ICD-9-CM code 410), coronary heart disease (ICD-9-CM codes 402, 410–414, and 429.2), diseases of the heart (ICD-9-CM codes 390–398, 402, 404, and 410–429), or cardiovascular disease (ICD-9-CM codes 390–448). ICD-9-CM indicates *International Classification of Diseases, Ninth Revision, Clinical Modification* (6).
